# Mechanistic understanding of female reproductive aging based on the chicken model

**DOI:** 10.1186/s40104-026-01435-6

**Published:** 2026-06-07

**Authors:** Junnan Zhang, Mingyue Gao, Congjiao Sun, Ning Yang

**Affiliations:** 1 State Key Laboratory of Animal Biotech Breeding, Frontier Science Center of Molecular Design Breeding, China Agricultural University, Beijing, 100193 China; 2 National Engineering Laboratory for Animal Breeding, College of Animal Science and Technology, China Agricultural University, Beijing, 100193 China

**Keywords:** Aging, Female, Genetic mechanism, Laying hen, Reproductive capacity

## Abstract

Female reproductive aging is a fundamental biological process characterized by a progressive decline in ovarian function, oocyte quality, and endocrine homeostasis, ultimately leading to reduced fertility and increased susceptibility to age-related diseases. Accumulating evidence indicates that reproductive aging is not merely a passive consequence of time but rather a tightly regulated process governed by complex genetic, epigenetic, and metabolic mechanisms. However, mechanistic dissection and translational exploration of female reproductive aging remain constrained by the limited availability of suitable animal models that faithfully recapitulate the human reproductive trajectory. In this review, we synthesize the current advances in understanding the molecular regulatory networks underlying female reproductive aging, with particular emphasis on key signaling pathways, cellular senescence, epigenetic regulation, hormonal control, and mitochondrial dysfunction coupled with oxidative stress. We highlight how the dysregulation of these interconnected mechanisms contributes to ovarian reserve depletion, follicular atresia, and declining oocyte competence across species. We propose that laying hens are a powerful and underutilized model for studying female reproductive aging. Laying hens exhibit a well-defined and highly reproducible reproductive lifespan characterized by distinct phases of peak and declining reproductive output, closely paralleling the age-related fertility decline in women. At the molecular level, hens share conserved regulatory features with humans, including hormonal signaling via the hypothalamic–pituitary–ovarian axis, age-associated oxidative stress, mitochondrial dysfunction, and epigenetic modulation of reproductive tissues. The daily ovulation cycle, measurable reproductive output, and responsiveness to metabolic and environmental interventions in hens further facilitate high-resolution and high-throughput investigations into aging-related mechanisms. By integrating evidence from human studies, mammalian models, and avian systems, this review highlights the translational value of laying hens in elucidating conserved genetic and epigenetic drivers of female reproductive aging. We discuss the current limitations and future perspectives for cross-species validation and multi-omics integration, aiming to facilitate the identification of actionable targets for delaying reproductive aging and improving female reproductive health.

## Introduction

**Fig. 1:**
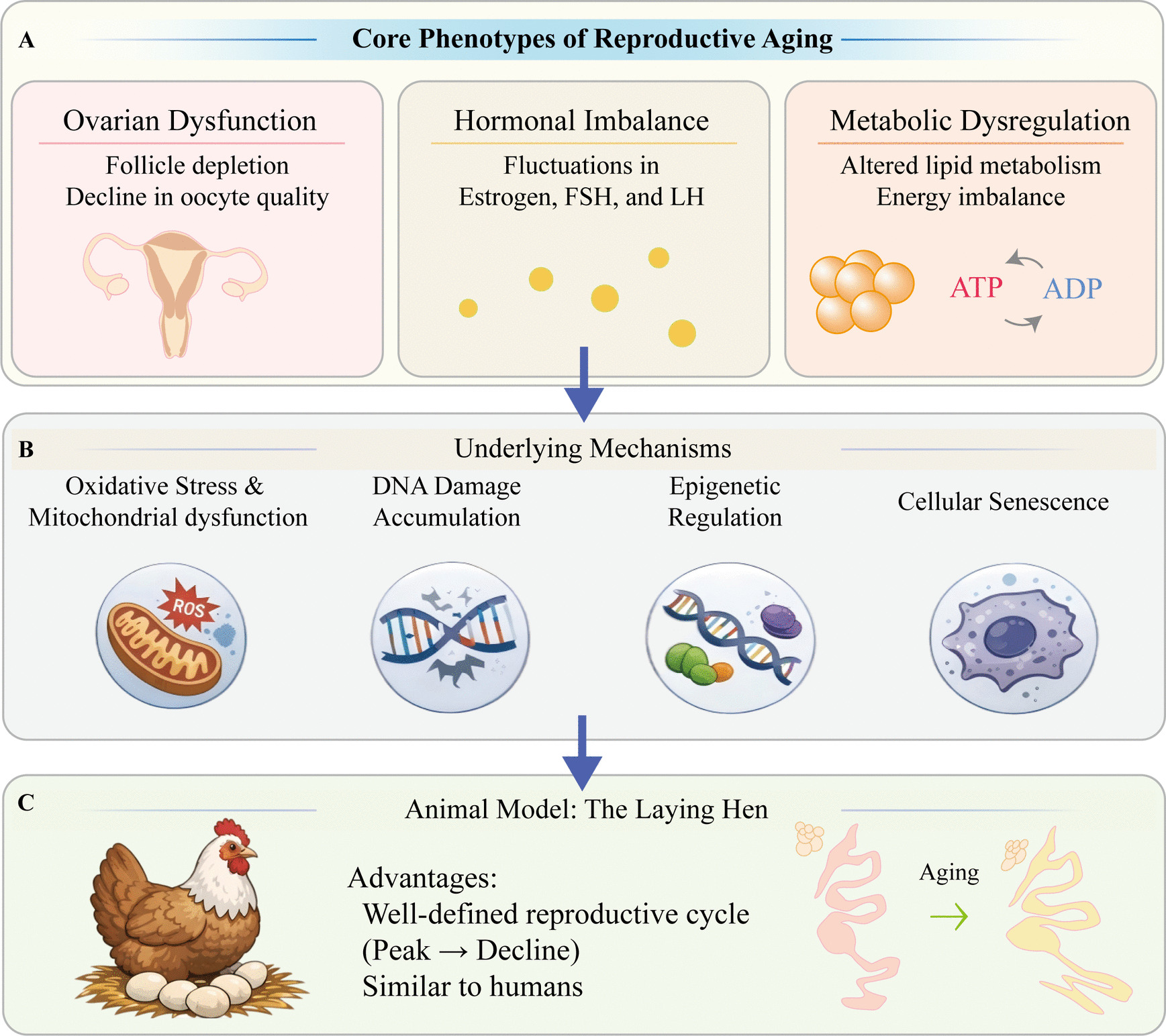
Core phenotypes and underlying mechanisms of reproductive aging. **A** Core phenotypes of reproductive aging (human). These include ovarian dysfunction, hormonal imbalance, and metabolic dysregulation. **B** Key underlying cellular and molecular mechanisms driving reproductive aging (human). **C** The laying hen (Gallus gallus) as a relevant animal model for studying reproductive aging

Human females reproductive aging is a complex biological process with profound implications for fertility and health. As age advances, the reproductive system undergoes significant changes, including ovarian dysfunction, decline in oocyte quality, and hormonal imbalance [1]. These alterations not only directly impair fertility but also indirectly compromise reproductive health through mechanisms such as lipid metabolism and energy balance. Ovarian dysfunction is central to reproductive aging and is characterized by the depletion of ovarian follicle reserves and reduction in oocyte quality. This often manifests as diminished ovulatory capacity and impaired oocyte developmental potential. Additionally, fluctuations in reproductive hormones such as estrogen, luteinizing hormone (LH), and follicle-stimulating hormone (FSH) exacerbate the deterioration of reproductive function. Concurrently, changes in lipid metabolism, including hepatic fat reserves and abdominal fat accumulation, disrupt energy homeostasis, further affecting reproductive performance (Fig. 1A).

Human reproductive aging involves intricate genetic and epigenetic regulatory mechanisms [2]. Key processes, including oxidative stress, mitochondrial dysfunction, and inflammatory signaling, significantly contribute to the decline in oocyte quality and ovarian function. Recent research has identified crucial genes and pathways, including those involved in DNA damage repair, energy metabolism, and hormonal regulation, as being central to the reproductive aging process [3]. However, translating these findings into effective interventions requires robust animal models that recapitulate human reproductive aging and allow controlled experimentation (Fig. 1B).

Laying hens have emerged as a valuable animal model because of their unique physiological characteristics. With a well-defined life cycle and distinct reproductive phases, including peak egg production and subsequent decline, laying hens provide an ideal model for investigating the mechanisms of reproductive aging. Moreover, their sensitivity to environmental and metabolic interventions presents an opportunity to explore the interplay between reproductive functions and systemic metabolism. This model allows researchers to examine not only the direct mechanisms of ovarian aging but also the complex relationship between lipid metabolism and reproductive health (Fig. 1C).

This review aims to provide a comprehensive overview of the genetic and epigenetic regulatory mechanisms underlying female reproductive aging, with a specific focus on insights gained from laying hen model. We will explore the direct and indirect effects of aging on the female reproductive system, evaluate the genomic and epigenetic changes associated with aging, and discuss the advantages and limitations of laying hens as a model organism in comparison with other animal models. By integrating findings from poultry science, genomics, and reproductive biology, this review aims to enhance our understanding of reproductive aging and identify potential strategies for preserving reproductive function and mitigating age-related fertility decline.

## Definition and manifestations of female reproductive aging

Female reproductive aging is a multifaceted biological process characterized by the progressive decline in ovarian function, marked by diminished oocyte quantity and quality, hormonal fluctuations, and eventual the cessation of menstruation, known as menopause [4, 5]. This phenomenon is distinct from somatic aging, as it follows a unique trajectory influenced by intrinsic and extrinsic factors, including genetic predispositions, environmental exposures, and lifestyle choices [6, 7]. The process begins prenatally, with the peak number of oocytes established before birth, followed by a continuous decline that accelerates as women approach menopause [8]. The onset of menopause, typically occurring between 45 and 55 years of age, signifies the end of a woman’s reproductive capacity and is associated with a significant increase in the risk of age-related diseases, such as osteoporosis, cardiovascular disease, and cognitive decline [9, 10].

A manifestations of female reproductive aging is the decline in ovarian reserve, which quantified clinically by biomarkers such as anti-mullerian hormone and FSH levels [11]. These biomarkers provide valuable insights into the remaining follicular pool and can help predict the timing of menopause [12]. Additionally, oocyte aging is associated with chromosomal instability, mitochondrial dysfunction, and telomere attrition, which contribute to reduced fertility and increased rates of miscarriage and aneuploidy in women of advanced maternal age [13, 14]. The decline in ovarian function also leads to hormonal imbalances, particularly a reduction in estrogen levels, which play a critical role in maintaining homeostasis across various physiological systems [15]. The loss of estrogen has widespread systemic effects, impacting bone density, cardiovascular health, and cognitive function, thereby contributing to the increased morbidity observed in postmenopausal women [16, 17].

## Mechanism of reproductive aging

Understanding the mechanisms of female reproductive aging is crucial, as the decline in reproductive capacity is often accompanied by an increased risk of age-related diseases and a reduced quality of life [4]. Recent research has highlighted the role of genetic pathways, epigenetic modifications, cellular senescence, hormonal regulation, mitochondrial function, and oxidative stress in driving ovarian aging, offering new insights into potential interventions to mitigate its effects [18, 19].

### Key signaling pathway

**Fig. 2:**
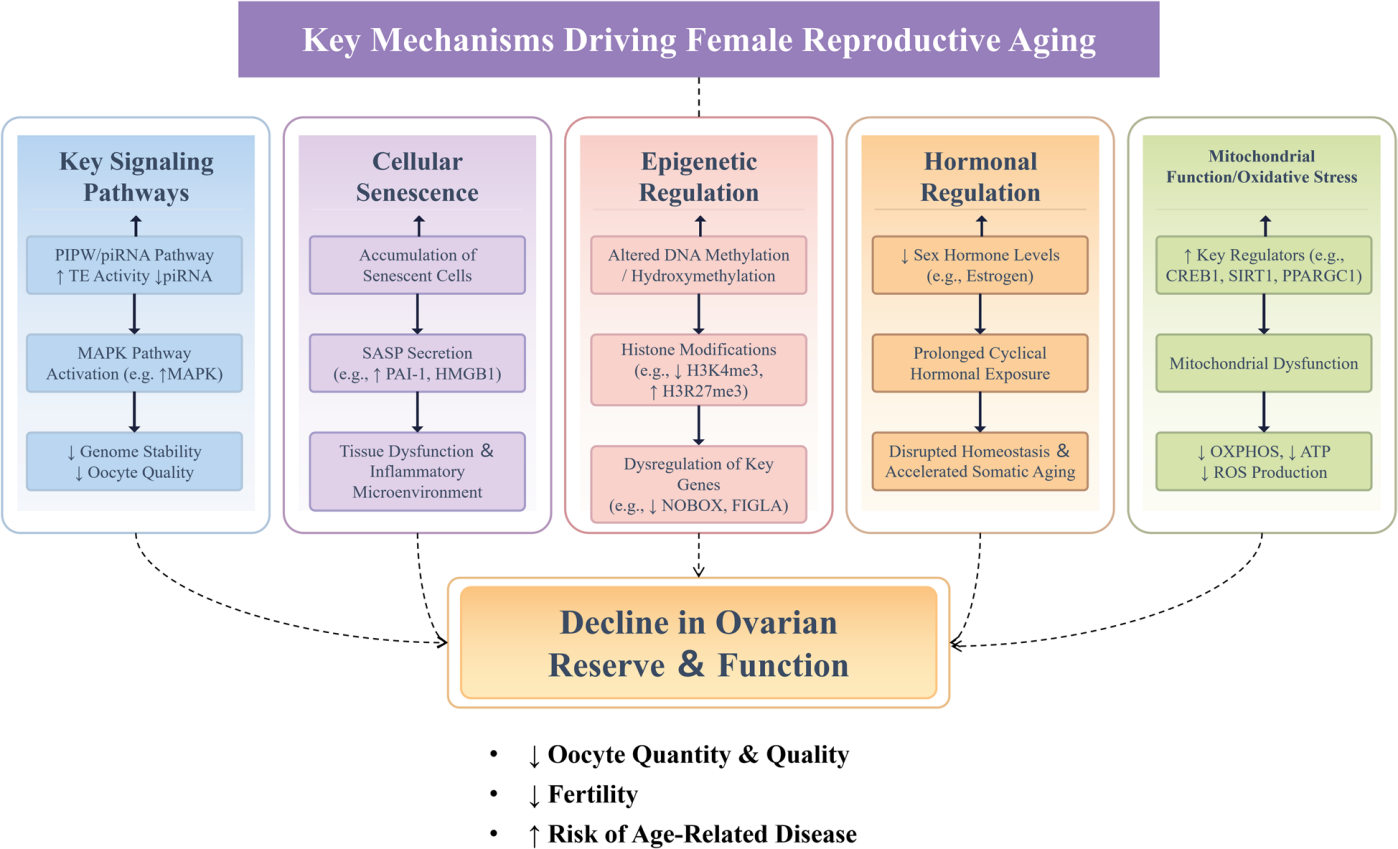
Key mechanisms driving female reproductive aging. This figure summarizes the core pathways involved in ovarian aging: (1) dysregulated signaling (PIWI/piRNA, MAPK), leading to genomic instability; (2) cellular senescence and SASP secretion (e.g., PAI-1, HMGB1), promoting inflammation; (3) epigenetic alterations (DNA/histone modifications), affecting genes such as *NOBOX* and *FIGLA*; (4) hormonal decline and cyclical exposure, disrupting homeostasis; and (5) mitochondrial dysfunction and oxidative stress, driven by regulators such as *CREB1*, *SIRT1*, and *PPARGC1*, reducing ATP and increasing ROS. Collectively, these mechanisms contribute to the decline in oocyte quality and ovarian reserve. ↑ indicates upregulation, ↓ indicates downregulation

In chickens, reproductive aging is characterized by a progressive decline in egg production, deterioration of eggshell quality, and reduced ovarian follicular function. Although the molecular mechanisms underlying these changes are not yet fully elucidated, emerging evidence suggests that conserved genetic pathways involved in genome stability and oocyte quality regulation play critical roles [20]. One such pathway is the PIWI/piRNA pathway, which is essential for maintaining genome integrity by suppressing transposable elements. In avian species, including chickens, PIWI genes and piRNAs have been identified in germ cells, indicating a conserved role in safeguarding genomic stability during oogenesis [21]. Although direct evidence linking PIWI/piRNA dysfunction to reproductive aging in chickens remains limited, studies in other model organisms provide important insights. For example, in the African turquoise killifish (*Nothobranchius furzeri*) have shown that PIWI pathway components are transiently downregulated in middle-aged ovaries, leading to elevated TE transcription and reduced piRNA levels, which may contribute to declining egg quality even before overt signs of aging are evident [22]. This suggests that the PIWI/piRNA machinery is essential for preserving ovarian function and that its dysregulation may serve as an early marker of reproductive aging. Similarly, in *Drosophila melanogaster*, increased expression of the piRNA machinery in aged reproductive tissues indicates an age-related investment in the maintenance of genome stability, further underscoring the importance of this pathway in reproductive aging [23]. The mitogen-activated protein kinase (MAPK) signaling pathway has also emerged as a key regulator of oocyte quality and reproductive aging. Another important regulatory pathway is the MAPK signaling pathway, which has been implicated in follicular development and oocyte maturation in chickens. Previous studies have demonstrated that MAPK signaling is actively involved in granulosa cell proliferation and differentiation in chicken ovaries, which are essential for follicular maturation and ovulation [24]. For instance, studies have demonstrated that MAPK activation in cumulus cells is necessary for gonadotropin-induced germinal vesicle breakdown (GVBD) during oocyte maturation, although its role in spontaneous GVBD remains dispensable [25]. Moreover, in *Caenorhabditis elegans*, activation of the MAPK pathway, particularly through the kinase MPK-1, is associated with a decline in oocyte quality with aging. Genetic and pharmacological manipulation of MPK-1 levels has shown that reduced MAPK signaling enhances oocyte quality, whereas increased signaling accelerates its deterioration [19]. These findings suggest that modulating MAPK signaling may be a potential therapeutic approach to mitigate the effects of reproductive aging (Fig. 2).

### Cellular senescence

Another critical aspect of female reproductive aging is cellular senescence, characterized by the accumulation of senescent cells in the ovaries (Fig. 2). In aged laying hens, increased oxidative damage, apoptosis, and impaired proliferation of granulosa cells have been reported, indicating a decline in cellular function within the ovarian microenvironment [26]. And senescent cells accumulate lipofuscin aggresomes and exhibit mitochondrial calcium dysregulation, impairing energy metabolism and oocyte quality [18]. In mice, ovarian aging is associated with the accumulation of senescent cells exhibiting a senescence-associated secretory phenotype (SASP), which promotes chronic inflammation and tissue dysfunction. Transcriptomic analyses have revealed increased expression of cyclin-dependent kinase inhibitors, such as *CDKN1A* and *CDKN2A*, as well as SASP-related factors including PAI-1 and HMGB1 [18]. These changes contribute to a deteriorated ovarian niche and impaired follicular development. Therefore, targeting cellular senescence and its associated secretory phenotype may represent a promising strategy to delay reproductive aging and improve laying persistence in chickens.

### Epigenetic regulation

Epigenetic regulation also plays a significant role in the aging of the female reproductive system (Fig. 2). Recent studies have elucidated how DNA methylation, histone modifications, and non-coding RNAs collectively influence the aging trajectory of reproductive tissues. For instance, hydroxymethylation, a modification associated with active DNA demethylation, is relatively abundant in the ovary and may play a role in regulating gene expression during aging. In chicken, H3K27ac and H3K4me1/2/3 dynamically modulate chromatin accessibility at promoters of key genes such as *DHCR7*, a critical enzyme in cholesterol synthesis linked to follicular selection. Estrogen-mediated epigenetic activation of *DHCR7* via histone acetylation (H3K27ac, H4K16ac) and methylation (H3K4me1/2) exemplifies how hormonal signals integrate with chromatin remodeling to sustain ovarian function during early aging stages [27]. And miR-210a-5p was found to suppress *RASL11B*, a GTPase that activates the MAPK pathway, thereby accelerating cellular aging when dysregulated [28]. In humans, the repression of transcription factors such as NOBOX and FIGLA, which are crucial for maintaining primordial follicle quiescence, has been observed in aging ovaries, further linking epigenetic changes to the decline in ovarian reserve [18]. Moreover, the interplay between DNA methylation and other epigenetic modifications, such as histone modifications and non-coding RNAs, adds another layer of complexity to ovarian aging. For example, age-related changes in histone methylation, including trimethylation of H3K4 and H3K27, have been implicated in the transcriptional regulation of genes involved in folliculogenesis and oocyte maturation [29]. Similarly, the dysregulation of microRNAs (miRNAs) and long non-coding RNAs (lncRNAs) has been linked to altered gene expression profiles in aging ovaries, suggesting a multifaceted epigenetic network governing ovarian function [30]. The construction of competing endogenous RNA networks has further elucidated the regulatory interactions between coding and non-coding RNAs, providing a comprehensive framework for understanding the molecular mechanisms underlying ovarian aging [30].

### Hormonal regulation

Hormonal regulation and the interplay between reproductive and somatic aging are critical factors in female reproductive aging (Fig. 2). The intricate hormonal regulatory mechanisms in chickens exhibit remarkable parallels with those in humans, particularly in the context of reproductive physiology and neuroendocrine control. Central to this similarity is the hypothalamic-pituitary-ovarian (HPO) axis, which governs reproductive functions in both species through conserved molecular pathways. In chickens, as in mammals, gonadotropin-releasing hormone (GnRH) serves as the pivotal regulator of LH and FSH secretion from the pituitary, orchestrating gonadal steroidogenesis and follicular development [31, 32]. However, the decline in ovarian reserve and the onset of menopause are associated with reduced levels of sex hormones in human, which disrupt homeostasis and accelerate somatic aging [4]. Experimental evidence in mice has shown that blocking ovarian cyclicity can reverse age-related changes in the myometrial transcriptome, suggesting that hormonal manipulation could be a strategy to mitigate uterine aging [33].

### Mitochondrial function and oxidative stress

Mitochondrial function and oxidative stress are central to the genetic regulation of ovarian aging, a process characterized by the decline in both the quantity and quality of oocytes, leading to diminished fertility in women (Fig. 2). Mitochondria, as the primary energy producers in cells, play a pivotal role in maintaining oocyte health and developmental competence. In chickens, aging oocytes exhibit significant downregulation of mitochondrially encoded subunits of respiratory chain complexes, which are critical for electron transport and adenosine triphosphate (ATP) synthesis [34, 35]. Concurrently, oxidative stress disrupts redox balance, damaging lipids, proteins, and DNA, while the downregulation of antioxidant genes (e.g., Gsto2, Msrb1) in aged oocytes diminishes cellular defenses, perpetuating a vicious cycle of oxidative damage and mitochondrial decay [36, 37]. Recent studies in chicken models prove that aged chicken oocytes display reduced mtDNA copy numbers and aberrant mitochondrial morphology, akin to findings in murine and primate models, suggesting evolutionary conservation of these mechanisms [34, 38]. In human, with advancing maternal age, mitochondrial dysfunction becomes increasingly evident, marked by reduced oxidative phosphorylation, diminished ATP production, and elevated levels of reactive oxygen species (ROS) [34, 37, 39]. These alterations not only compromise the bioenergetic capacity of oocytes but also contribute to the accumulation of oxidative damage, which further exacerbates aging. For instance, studies have shown that aged oocytes exhibit significant reductions in mtDNA content and impaired mitochondrial dynamics, such as decreased mitochondrial coverage and altered morphology, which are associated with increased ROS production and oxidative stress [36, 40].

The interplay between mitochondrial dysfunction and oxidative stress is mediated through several genetic and molecular pathways. For example, the transcription factor *CREB1* has been identified as a key regulator of mitochondrial biogenesis and function in granulosa cells, which are critical for supporting oocyte development. Downregulation of *CREB1* leads to reduced expression of bioenergetic-related genes, such as *PRKAA1* and *PRKAA2*, and impairs mitochondrial function, thereby accelerating granulosa cell senescence and oocyte aging [35]. Additionally, the cofactors SIRT1 and PPARGC1A, which are involved in mitochondrial biogenesis, are downregulated in aging granulosa cells, further contributing to mitochondrial dysfunction and oxidative stress [41]. These findings underscore the importance of *CREB1* and its associated pathways in maintaining mitochondrial health and delaying ovarian aging.

In conclusion, although much of the mechanistic understanding of reproductive aging has been derived from mammalian models, accumulating evidence indicates that these pathways are evolutionarily conserved and highly relevant to chickens. Integrating chicken-specific data with insights from other species will be essential for advancing our understanding of reproductive aging and for developing strategies to improve reproductive longevity in laying hens.

## Molecular and physiological changes in reproductive capacity of hens across lifespan

### Laying cycle of hens

The entire life span of hens is a complex physiological process characterized by distinct stages: the early, peak, and late laying periods. Each stage is marked by unique physiological, metabolic, and behavioral changes that significantly influence the reproductive capacity. Understanding these changes is critical for optimizing management practices and extending the productive lifespan of laying hens [42, 43].

The early laying period, which typically begins at 18–22 weeks of age, is marked by the onset of sexual maturity and initiation of egg production. During this phase, increasing levels of circulating estrogen stimulate the formation of medullary bone, which serves as a calcium reservoir for eggshell formation [44]. The development of the reproductive system, including the ovary, oviduct, and follicles, is critical at this stage. The peak laying period, which typically occurs between 23 and 33 weeks of age, is characterized by maximum egg production. During this phase, hens exhibit high metabolic activity to support the daily laying of eggs. The late laying period, after 72 weeks of age, is marked by a significant decline in egg production. This phase is particularly challenging for the poultry industry, as hens experience physiological aging, reduced calcium mobilization, and increased susceptibility to metabolic disorders, such as osteoporosis and fatty liver disease [44, 45].

### The regulation of reproductive capacity with aging in laying hens

The decline in the reproductive capacity of laying hens with increasing age is influenced by multifaceted genetic regulatory mechanisms. As hens age, ovarian aging manifests through a series of physiological changes, including reduced follicular development, decreased yolk precursor synthesis, and altered endocrine profiles, all of which contribute to diminished egg-laying performance [46]. Transcriptomic analyses have revealed that aging hens exhibit significant downregulation of genes critical for yolk precursor formation, such as those involved in lipid synthesis and antioxidant defense mechanisms in the liver, which are essential for maintaining a high egg production rate. Additionally, the expression of estrogen receptors and genes associated with steroidogenesis, such as *CYP19A1* and *STAR*, declines with age, further impairing follicular development and ovulation [47].

One key genetic factor contributing to this decline is the dysregulation of granulosa cell (GC) function, which plays a pivotal role in follicular development and atresia. In aging hens, GCs exhibit increased apoptosis and reduced proliferation, driven by the upregulation of pro-apoptotic genes such as *CASP3* and the downregulation of cell cycle regulators such as *CDK2* and *CCND1* [20, 47]. This cellular deterioration is exacerbated by age-related DNA damage, which activates the CHK2/p53 pathway, leading to cell cycle arrest and apoptosis in prehierarchical follicles [20]. Another critical aspect of the genetic regulation of reproductive capacity is the HPO axis, which orchestrates reproductive functions through the complex interplay of hormones and neuropeptides. Aging hens exhibit altered expression of genes within the HPO axis, such as *GNRH*, *GNRHR*, and *CYP11A1*, which disrupt the hormonal balance necessary for follicular development and ovulation [20]. In conclusion, the decline in the reproductive capacity of aging hens is governed by a network of genetic regulatory mechanisms, including the dysregulation of granulosa cell function and hormonal imbalances within the HPO axis. Understanding these mechanisms provides valuable insights into the molecular basis of reproductive aging in poultry species.

### The similarities in reproduction between laying hens and humans

The reproductive aging processes in laying hens and women exhibit striking parallels, offering a unique model for studying the biological mechanisms underlying age-related declines in fertility and reproductive efficiency. Both species experience a gradual reduction in reproductive capacity as they age, which is characterized by diminished ovarian function, hormonal imbalances, and increased oxidative stress. In humans, females experience a decline in fertility after the age of 37, culminating in menopause, which is driven by a reduction in ovarian follicular reserve and oocyte quality. Similarly, in laying hens, reproductive aging manifests as a decline in egg production during the late laying cycle, primarily due to ovarian aging, reduced yolk precursor synthesis, and decreased estrogen levels [48]. These shared features highlight the utility of laying hens as a model for understanding human reproductive aging, particularly in elucidating the molecular and physiological pathways involved.

One of the key similarities is the role of oxidative stress in driving reproductive senescence. In laying hens, oxidative stress accumulates with age, leading to granulosa cell dysfunction, follicular atresia, and reduced yolk precursor formation in the liver. This is mirrored in women, where oxidative stress contributes to oocyte damage, mitochondrial dysfunction, and a decline in oocyte quality. Both species exhibit a diminished capacity to counteract oxidative stress owing to age-related declines in antioxidant enzyme activity, underscoring the importance of oxidative damage in reproductive aging. Interventions targeting oxidative stress, such as the administration of antioxidants, such as lycopene in hens, have shown promise in ameliorating ovarian aging and improving reproductive outcomes [26]. In humans, vitamin C exerts multiple protective effects on ovarian cells by activating the key antioxidant transcription factor NRF2. These effects include delaying aging, inhibiting inflammation, maintaining chromatin stability, and enhancing mitochondrial function [49]. Hormonal regulation also plays a critical role in reproductive aging in both species. In laying hens, estrogen levels peak during the early laying period but decline significantly with age, leading to reduced yolk precursor synthesis and egg production [50]. Similarly, women experience a decline in estrogen levels during perimenopause, which contributes to the cessation of ovulation and the onset of menopause [51].

Despite these similarities, notable differences exist between the reproductive biology of laying hens and that of women. For instance, hens exhibit reproductive plasticity through molting, a process that temporarily halts egg production and rejuvenates the reproductive tract, leading to improved laying efficiencies. While humans do not undergo an analogous process, understanding the mechanisms underlying molting could provide insights into potential interventions to restore reproductive function in aging females. Additionally, the ability of laying hens to produce eggs daily offers a unique and reliable assay to study reproductive aging, providing a high-throughput model for evaluating anti-aging treatments.

## Common age-related diseases in chickens and corresponding human disease models

### Osteoporosis and osteoarthritis

Age-related skeletal disorders, such as osteoporosis and osteoarthritis, are prevalent in aging chickens and share mechanistic parallels with human conditions. The bipedal nature of chickens subjects their tibio-femoral joints to mechanical stresses akin to humans, making them an ideal model for studying load-bearing joint degeneration. Chronic inflammatory conditions, such as *Eimeria *spp. infections, disrupt bone and cartilage homeostasis in chickens, leading to reduced trabecular bone volume, increased trabecular separation, and articular cartilage thinning-phenotypes reminiscent of post-infectious osteopathies in humans. The systemic inflammation driven by parasitic infections elevates pro-inflammatory cytokines (e.g., IL-1β, IL-6) and osteoprotegerin, while reducing insulin-like growth factor 1 (IGF-1), mirroring the inflammatory bone loss observed in metabolic syndrome-associated osteoporosis [52].

### Cardiovascular diseases and metabolic syndrome

Chickens are increasingly recognized as models for cardiovascular diseases and metabolic syndrome, given their susceptibility to diet-induced obesity, dyslipidemia, and insulin resistance. The high cholesterol and low choline diet model in chickens induces hyperlipidemia, hypercholesterolemia, and hepatic steatosis, closely mimicking human non-alcoholic fatty liver disease (NAFLD) and its cardiovascular complications. These metabolic disturbances are associated with elevated triglycerides and cholesterol, akin to the dyslipidemia observed in human metabolic syndrome. The chicken model also offers insights into the interplay between NAFLD and extrahepatic disorders, such as cardiovascular dysfunction, by enabling the study of comorbid conditions like viral hepatitis (e.g., HEV) and systemic inflammation [53].

### Spontaneous tumors

Chickens exhibit spontaneous ovarian cancers that closely mimic human ovarian cancer in terms of histopathology and molecular signatures, making them an invaluable tool for investigating tumor and therapeutic interventions [54]. Furthermore, using chickens for liver cancer research is also a hotspot, which is an important approach for studying metabolic and inflammatory diseases. Recent studies have demonstrated that microplastics, an environmental pollutant, disrupt this axis in chickens, leading to intestinal barrier dysfunction, dysbiosis, and subsequent hepatic lipid metabolism disorders—a process analogous to non-alcoholic fatty liver disease and hepatocellular carcinoma in humans [55]. This model not only elucidates the mechanisms of pollutant-induced carcinogenesis but also provides a platform for testing interventions aimed at mitigating liver damage. Finally, the chicken model also excels in preclinical oncology research, particularly in evaluating immunotherapy and drug efficacy. The chorioallantoic membrane assay has been validated as a cost-effective, 3R-compliant alternative to rodent models for screening PD-1/PD-L1 inhibitors, with pembrolizumab demonstrating cross-species efficacy in blocking immune checkpoints and restoring T-cell cytotoxicity [56].

## Translational implications of the laying hen model for human reproductive health

### Therapeutic targets for delayed aging validated in laying hen research

The laying hen has emerged as a uniquely valuable model for studying reproductive aging, offering translational insights into human reproductive health due to its exceptional reproductive efficiency, plasticity, and shared physiological pathways with mammals [48, 57, 58]. Unlike mammalian models, hens exhibit a daily ovulation cycle, producing an egg nearly every 24 h, which provides a high-resolution system for investigating age-related declines in reproductive function [46]. Key parallels include diminished estrogen signaling (reduced serum 17β-estradiol and estrogen receptor expression), oxidative stress in metabolic organs like the liver, and dysregulation of yolk precursor synthesis (e.g., vitellogenin and apolipoproteins), analogous to impaired follicular development and steroidogenesis in aging human ovaries [59]. Furthermore, the hen’s ability to undergo molting-a controlled fasting protocol that temporarily rejuvenates reproductive tract function and restores peak laying efficiency-provides a conceptual paradigm for understanding how temporary reproductive rest and metabolic reset may mitigate age-related reproductive decline. While humans do not undergo an analogous process, insights from molting may inform the development of interventions targeting metabolic regulation, oxidative stress, and reproductive rejuvenation [58]. Metabolomic studies in molted hens have revealed systemic rejuvenation, including reduced ‘metabolic noise’ and restored mitochondrial function, suggesting conserved mechanisms that may inspire human fertility preservation strategies, without implying direct translational application [23].

The hen model also elucidates the impact of circadian and metabolic disruptions on reproductive aging, with direct implications for human chrononutrition research. Nighttime eating in hens, despite negligible caloric contributions, accelerates reproductive decline by disrupting circadian rhythms, akin to shift-work-associated fertility impairments in women [57, 60]. This aligns with mammalian data showing that time-restricted feeding improves metabolic health and longevity, underscoring the translational potential of dietary synchronization for mitigating age-related infertility [60, 61]. Additionally, the linear cascade governing hen ovulation, where failure at any step abruptly halts reproduction, resembles the fragility of human folliculogenesis, highlighting the shared vulnerabilities of reproductive systems under aging pressures.

The hen model leverages advanced multi-omics approaches to identify conserved biomarkers. Transcriptomic analyses of uterine, liver, and intestinal tissues reveal age-related dysregulation in genes critical for eggshell formation (e.g., *FGF14*, *COL25A1*) and nutrient metabolism, paralleling human endometrial and hepatic dysfunction during aging [46, 59, 62]. Notably, lncRNAs, such as TCONS_00181492, modulate eggshell quality deterioration, offering a novel framework for studying the epigenetic regulators of reproductive aging in vertebrates [62]. The model’s scalability further enables high-throughput screening of anti-aging interventions, such as phytase supplementation, to optimize phosphorus metabolism, a strategy relevant to human bone health during menopause [63].

For human applications, the hen reproductive tract provides a tractable system for testing pharmacological or dietary interventions targeting shared pathways (e.g., estrogen signaling and oxidative stress) before clinical trials [59]. Its rapid aging trajectory and quantifiable output (egg production) accelerate translational research, while metabolic noise reduction post-molting suggests a universal biomarker for assessing rejuvenation therapies [23]. Future directions include exploring the hen’s germline epigenetics, where heterochromatin stability contrasts with somatic aging patterns, to uncover the mechanisms that preserve reproductive longevity [23]. By bridging avian and mammalian biology, the laying hen model not only advances poultry science but also pioneers actionable strategies to combat human reproductive aging, from chrononutrition to targeted epigenetic therapies [62].

In conclusion, the unique reproductive plasticity of laying hens, coupled with their physiological and molecular parallels to humans, positions them as an indispensable model for decoding and mitigating age-related fertility decline. Its integration into translational research pipelines promises to accelerate the development of interventions to extend reproductive spans in both agricultural and clinical settings in the future.

### Cross-species validation of candidate genes and therapeutic targets

Cross-species investigations of candidate genes and intervention targets in ovarian biology among humans, mice, and laying hens have unveiled conserved and divergent molecular mechanisms governing folliculogenesis, steroidogenesis, and reproductive pathologies. Recent advances in transcriptomics and functional genomics have identified critical pathways, such as the WNT/β-catenin signaling cascade, which is implicated in ovarian differentiation across vertebrates. For instance, RNA-seq analyses of embryonic chicken gonads have revealed sexually dimorphic expression of *FGFR3*, *CAPN5*, and *GPR56*, which are also associated with human ovarian development and disorders such as polycystic ovary syndrome [64]. Similarly, murine studies have highlighted the role of *FOXL2* in repressing male-specific genes and activating aromatase, a mechanism conserved in avian species in which *FOXL2* precedes *CYP19A1* expression during ovarian differentiation [64]. Technological innovations, such as *in ovo* electroporation in chickens and CRISPR-Cas9 in mice, have enabled the functional validation of candidate genes. For example, *RSPO1* gain-of-function models in mice have demonstrated its pivotal role in granulosa cell proliferation via WNT/β-catenin activation, a pathway that is also dysregulated in human ovarian tumors. Importantly, components of the WNT/β-catenin signaling pathway have also been identified in avian ovarian development, supporting the conservation of this regulatory mechanism across species [65]. However, comparative transcriptomic analyses have revealed species-specific differences in oocyte maturation processes. For instance, studies in mammals have shown divergent degradation patterns of maternal mRNAs during meiosis, with limited overlap in transcript dynamics between species [66]. These findings highlight that, while core regulatory pathways are conserved, species-specific regulatory mechanisms exist. Therefore, integrating data from laying hens with mammalian models may provide a more comprehensive understanding of human oocyte maturation and reproductive aging.

Intervention targets have emerged from studies on mitochondrial function and oxidative stress. In mice, Mcl-1 knockout disrupts oocyte viability by impairing mitochondrial respiration and increasing ROS levels, whereas Bax deletion rescues apoptosis, highlighting the therapeutic potential of modulating BCL-2 family proteins in age-related ovarian decline [67]. Similarly, avian models exposed to the aromatase inhibitor fadrozole exhibited gonadal sex reversal, implicating estrogen signaling as a target for manipulating reproductive phenotypes in poultry and potentially human [68]. However, challenges persist in translating findings across species due to differences in follicular architecture (e.g., single dominant follicle in humans vs. hierarchical follicles in hens) and hormonal regulation. Additionally, epigenetic studies on Alzheimer’s disease have revealed methylation changes in genes such as *SORL1* and *BIN1*, which are expressed in ovarian tissue, suggesting pleiotropic effects of neurodegenerative risk loci on reproductive aging [69]. Future directions include leveraging single-cell RNA-seq to dissect granulosa cell heterogeneity and developing organoid models to test the efficacy of cross-species gene editing. By bridging evolutionary biology and clinical research, these efforts could yield precision therapies for infertility and ovarian disorders, while optimizing poultry production through genetic selection [64, 66, 67]. In conclusion, cross-species research has illuminated both universal and species-specific ovarian mechanisms, with translational implications ranging from assisted reproductive technologies to conservation biology. However, the limited overlap in gene networks between humans and model organisms underscores the necessity of integrative approaches that combine functional assays, population genetics, and computational modeling to refine intervention strategies.

## Challenges and future perspectives

### Limitations of the laying hen model for translational research

**Fig. 3:**
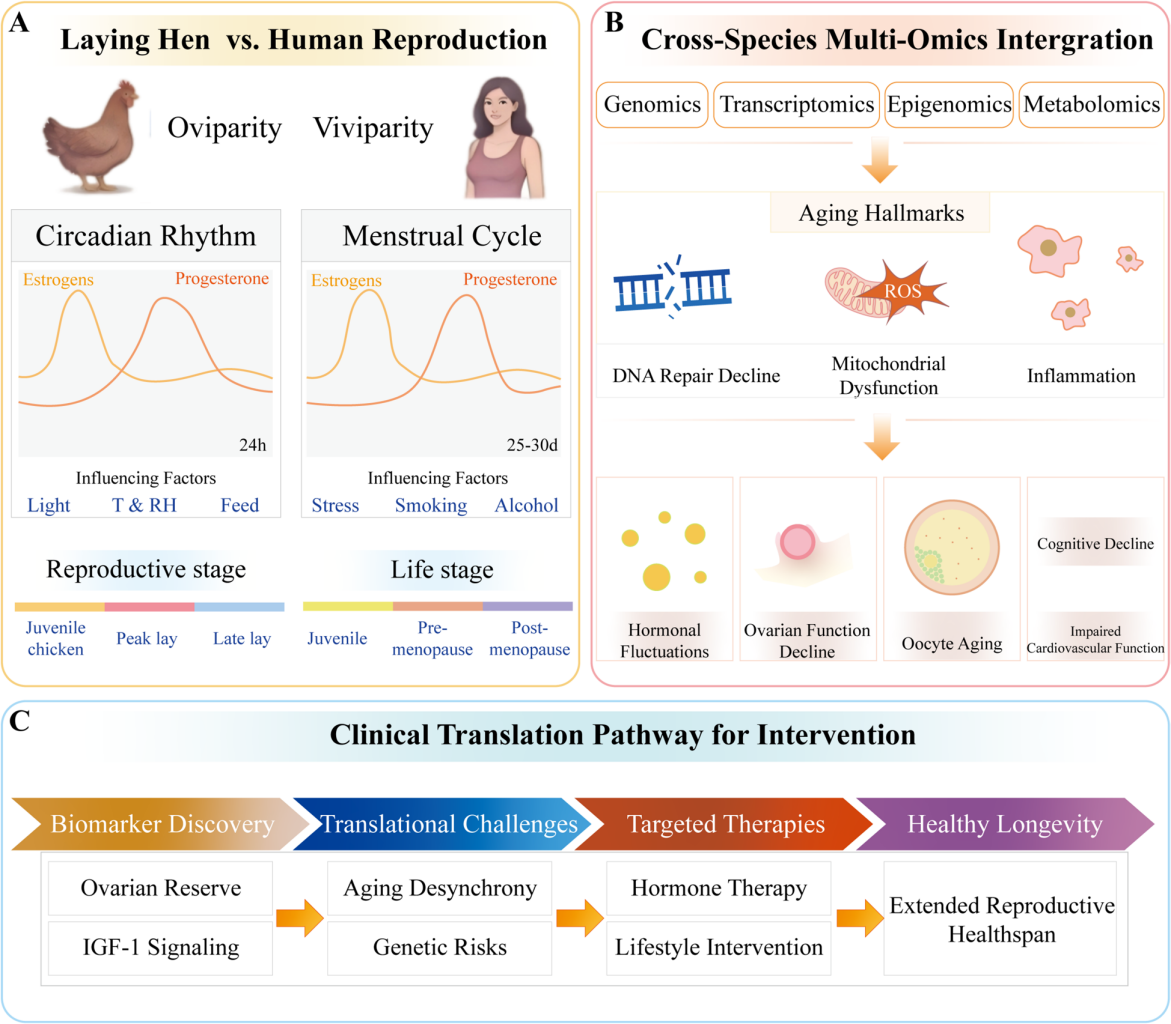
Comparative analysis of reproductive aging in laying hens and humans and a translational roadmap for intervention. **A** Schematic comparison of reproductive traits and aging patterns between oviparous (laying hens) and viviparous (humans) species. In laying hens, reproductive cycles are governed by circadian rhythms influenced by light, temperature, humidity, and feed, with egg-laying stages progressing from juvenile to peak and late lay. In humans, the menstrual cycle (~25–30 d) is regulated by estrogen and progesterone and is influenced by factors such as stress, smoking, and alcohol. Reproductive aging in humans spans from the juvenile stages to pre- and post-menopause, paralleling the decline in ovarian function. **B** Cross-species multi-omics integration framework for studying reproductive aging. Genomic, transcriptomic, epigenomic, and metabolomic data have been integrated to elucidate conserved aging hallmarks, including DNA repair decline, mitochondrial dysfunction, inflammation, hormonal fluctuations, ovarian function decline, oocyte aging, and impaired cardiovascular function. **C** Proposed clinical translation pathway for developing interventions against reproductive aging. The pathway includes biomarker discovery (e.g., ovarian reserve, IGF-1 signaling), identification of translational challenges (e.g., aging desynchrony, genetic risks), and development of targeted therapies (e.g., hormone therapy, lifestyle interventions) aimed at promoting healthy longevity and extending the reproductive healthspan

The application of research findings from the laying hen model to humans is characterized by both complexity and limitations. Throughout the long course of evolution, significant differences have emerged among species at multiple levels, including physiological structure and reproductive strategies. Due to physical structural constraints, the egg-laying hen model is challenging to use for studying the regulatory mechanisms of the human menstrual cycle. In the regular ovulatory menstrual cycle, steroid hormones in women exhibit cyclical fluctuations. Despite individual variations, a 28-day cycle is generally considered the standard, encompassing the early follicular phase (where estrogen and progesterone levels are relatively low), late follicular phase (characterized by elevated estrogen and low progesterone levels), and mid-luteal phase (where both estrogen and progesterone levels are high). Research indicates that lifestyle factors, such as stress, smoking, and alcohol consumption, can significantly impact menstrual regularity and fertility [70, 71] (Fig. 3A).

Another important limitation in the transformation process arises from the fundamental differences in reproductive strategies between mammals and poultry, namely, the distinction between viviparity and oviparity [72]. Viviparity refers to the reproductive mode in which the embryo develops within the reproductive tract of the mother and is ultimately born as a live offspring [73]. In contrast, oviparity is a reproductive mode in which females lay eggs. The evolutionary transition from oviparity to viviparity is a highly complex process that involves numerous changes in anatomy, physiology, behavior, and genetics [74, 75]. Currently, comparative studies on viviparity in mammals versus oviparity in other groups remain relatively limited, partly because of the high complexity of the evolutionary pathways of both.

Nevertheless, despite these limitations, certain physiological characteristics of laying hens still provide complementary value for translational research. For example, chickens exhibit a diurnal activity pattern and a well-defined 24-h circadian rhythm, which is comparable to that of humans [76, 77]. This makes them a useful model for investigating the interactions between circadian regulation, reproduction, and aging [57]. However, these advantages do not fully overcome the fundamental differences in reproductive biology between avian and mammalian species.

### Current status and advances in cross-species multi-omics integration

The integration of multi-omics approaches has revolutionized our understanding of the genetic and molecular mechanisms underlying female reproductive aging resistance, offering unprecedented insights into conserved and species-specific regulatory networks (Fig. 3B). Recent advancements in single-cell and bulk RNA sequencing, chromatin accessibility profiling, and metabolomics have enabled cross-species comparisons, revealing shared pathways such as DNA repair, mitochondrial dysfunction, and inflammatory responses that are critical in ovarian aging [35, 78, 79]. For instance, transcriptomic analyses in mice and humans have identified age-dependent declines in DNA repair genes (e.g., *Rif1* and *Paxip1*) and mitochondrial-encoded oxidative phosphorylation components, which correlate with oocyte quality deterioration. These findings underscore the utility of cross-species integration in identifying conserved hallmarks of reproductive aging. However, key challenges remain in reconciling species-specific differences. For example, while mice show linear ovarian follicle depletion, humans exhibit a more abrupt menopausal transition, necessitating the careful translation of findings [18, 80]. The non-obese diabetic/severe combined immunodeficient mouse model, which recapitulates human ovarian aging phenotypes (e.g., fibrosis and reduced mtDNA copy number), has proven valuable for testing interventions such as senolytics or plasma-based therapies [80, 81]. Cross-species meta-analyses also reveal divergent epigenetic regulation: unlike somatic tissues, ovarian aging in mice shows no global DNA hypomethylation but exhibits localized changes at transposable element loci, suggesting tissue-specific epigenetic clock [18, 79]. In conclusion, multi-omics integration across species has illuminated conserved genetic regulators of female reproductive aging, while highlighting the need for context-specific validation. By combining high-resolution molecular profiling with functional assay, this approach holds promise for identifying actionable targets to mitigate age-related fertility decline and improve healthy lifespan [22, 82, 83].

### Clinical translation pathway for anti-aging interventions

The clinical translation pathway for female anti-reproductive aging interventions represents a critical frontier in precision medicine, bridging the gap between mechanistic insights into ovarian senescence and actionable therapeutic strategies (Fig. 3C). Reproductive aging in women is characterized by a complex interplay of hormonal decline, follicular depletion, and systemic aging cascades, with menopause marking a pivotal transition associated with accelerated morbidity [4, 5]. Recent advances in biomarker discovery, including genetic markers of ovarian reserve and dynamic assessments of IGF-1 signaling in oocyte quality, have enabled the earlier identification of at-risk populations, paving the way for targeted interventions [84]. However, the translational pipeline must address key challenges: the desynchronization of reproductive and somatic aging trajectories [4], the 20% accelerated mutation rate in reproductive tissue [85], and the underutilization of preventive care in high-risk groups, such as BRCA mutation carriers [86, 87]. Emerging paradigms include hormone therapy optimization and integrative models combining Mediterranean diet adherence with supervised exercise to modulate the penetrance of hereditary cancer risks. By anchoring interventions in the biological nexus of reproductive decline, from follicular depletion to systemic inflammation, this translational framework promises to redefine healthy longevity for women globally.

## Conclusion

Female reproductive aging is a complex and tightly regulated process involving ovarian decline alongside systemic metabolic and endocrine alterations. Accumulating evidence highlights the coordinated roles of key signaling pathways, cellular senescence, epigenetic remodeling, hormonal dysregulation, and mitochondrial dysfunction in driving the progressive loss of oocyte quality and reproductive capacity. Despite significant advances, effective translational strategies remain limited, partly due to the lack of suitable experimental models. In this context, laying hens represent a valuable and underutilized model, exhibiting conserved features with women in ovarian aging, endocrine regulation, and oxidative stress dynamics. Although species-specific differences require careful interpretation, integrating insights from laying hens with mammalian and human studies provides a promising cross-species framework for advancing our understanding of reproductive aging and identifying potential therapeutic targets.

## Data Availability

Not applicable.

## Abbreviations

ATP: Adenosine triphosphate
FSH: Follicle-stimulating hormone
GC: Granulosa cell
GnRH: Gonadotropin-releasing hormone
GVBD: Gonadotropin-induced germinal vesicle breakdown
HPO: Hypothalamic-pituitary-ovarian
IGF-1: Insulin-like growth factor 1
LH: Luteinizing hormone
lncRNAs: Long non-coding RNAs
MAPK: Mitogen-activated protein kinase
NAFLD: Non-alcoholic fatty liver disease
ROS: Reactive oxygen species
SASP: Senescence-associated secretory phenotype
